# Prevalence of Lifestyle Risk Factors for Cardiovascular Diseases Among Adults in Taif City, Saudi Arabia: An Analytical Cross-Sectional Study

**DOI:** 10.7759/cureus.78338

**Published:** 2025-02-01

**Authors:** Faisal Abdulrahman Alotaibi, Fawaz Khalid Abdullatif Munshi, Zahran Majed Ayidh Alzahrani, Mohammed Nasser M Algahtani

**Affiliations:** 1 Preventive Medicine, Saudi Board of Community Medicine, Taif, SAU; 2 General Practice, Taif Health Cluster, Taif, SAU; 3 Medicine, Taif University, Taif, SAU; 4 Interventional Cardiology, King Abdulaziz Medical City, Riyadh, SAU

**Keywords:** adults, cardiovascular diseases, cross-sectional, lifestyle risk factors, taif

## Abstract

Introduction and aim: Cardiovascular diseases are a major cause of morbidity and mortality and are on the rise in developing countries, including Saudi Arabia. This analytical cross-sectional study aimed to identify the prevalence of such modifiable lifestyle risk factors (LRFs) for cardiovascular diseases (CVDs) among adults in Taif city, Kingdom of Saudi Arabia (KSA), where urbanization is taking place at a very fast pace.

Methods: In this case, 300 people responded to the survey that adopted a stratified random sampling method to recruit participants, where information was gathered about the participant’s demography, smoking profile, physical activity, dietary health, and knowledge about CVD risk factors.

Results: The majority of patients had multiple modifiable CVD risk factors as follows: physical inactivity (52%), unhealthy dieting (47% regularly consumed fried foods), obesity (25%), smoking history (55%), and hypertension history (34%). Subgroup analysis revealed that residence, education, income, and job-related stress were statistically significant concerning the number of CVD risk factors, which explains the rationale for targeted primary prevention strategies. Thus, the promotion of the campaigns of CVD symptoms and risk factors with modifications among the Taif city adult population has emerged as an imperative consideration for the improvement of health standards.

Conclusion: Thus, these findings point to the fact that the population in Taif City is at high risk for CVD attributed to urbanization, a sedentary lifestyle as well as the adjustment to a Westernized diet. These findings may be applied when formulating population-specific prevention interventions to address the epidemic burden of CVDs in this region. The research also strengthens the argument for the need to tackle modifiable risk determinants to reverse the increasing trends of cardiovascular diseases in Saudi Arabia.

## Introduction

Cardiovascular diseases (CVDs) are some of the most prevalent medical conditions and are a leading cause of death globally than any other disease [1]. In the past, it has been thought that cardiovascular diseases affected developing nations to a lesser extent. Nonetheless, the demands of cardiovascular diseases in developing countries have now reached epidemic levels [2]. Furthermore, cardiovascular diseases (CVDs) are ranked among the leading factors of morbidity and mortality and have also emerged as diseases of concern in most developing nations, including Saudi Arabia (KSA). Taif city is one of the large urban cities in Saudi Arabia that has undergone urbanization, and lifestyle changes could be associated with an increase in the rate of prevalence of CVDs. However, there is still scanty literature on the epidemiology and distribution of these lifestyle-related risk factors in this particular city. Awareness of the extent, nature, and distribution of behavioral risk factors for cardiovascular diseases, including smoking, physical inactivity, and poor diet, and their contribution to the overall burden are important to guide public health interventions. Further, the general knowledge about these risk factors and their significance in the prevention of CVD is still low. Concerning these factors in Taif city, this study endeavors to bridge the foregoing epidemiological gap and identify the current status of cardiovascular threat factors. The conclusions will help in the formulation of future public health interventions and the creation of school-based intervention and prevention programs that will meet the needs of the Taif people. An upsurge in the prevalence of hypertension and diabetes in the Middle Eastern and North African countries. For example, a qualitative study on hypertension in Saudi Arabia conducted by Aldiab et al. estimated the hypertension rate to be 25%, with regional variations observed, including a rate of 0% in some areas of Taif [3]. Tobacco use is still widely recognized as one of the biggest risk factors for CVDs. Studies conducted by Razzak et al. revealed that KSA has one of the highest smoking prevalence, especially among men, and this puts the population at higher risk of cardiovascular events [4]. Another risk factor is diet, which consists of high saturated fats, low vegetables, and fruits. In a study conducted by Al-Lahou et al., it was established that the traditional Saudi diet is linked with an increased number of cases of obesity and metabolic syndrome, which are leading factors to CVDs [5].

Awareness of CVD risk-determining factors is key to prevention. Several studies have been conducted in different developing countries, including Saudi Arabia, to determine the levels of awareness of the general populace concerning risk factors associated with CVD. For example, Almalki et al. noted that Saudi adults had low knowledge levels about CVD when receiving medical care, and a significant population remained oblivious to the consequences of certain lifestyles on the heart and blood vessels [6]. In addition, it is expected that the same cardiovascular diseases will rank among the leading causes of mortality in these countries within the next fifteen years. The current Asian and Middle Eastern populations seem to be candidates for cardiovascular risk factors that are abundant [1,7]. From the National Health Survey of Pakistan, it is estimated that hypertension and diabetes are present in 33% and 25% of individuals older than 45 years of age, respectively [8,9]. Also, the prevalence of smoking in men is 28%, which increases to 41% in the 40-to-49-year age range. In addition, nutritional preference in this population focuses on parameters that highlight the intake of saturated fats and ghee-hydrogenated vegetable oil [10]. Awareness of CVD and the potential risks for its occurrence is crucial (although not sufficient enough) for a person to modify their behavior to protect from cardiovascular diseases.

The main purpose of this research is to determine the level of adherence to the lifestyle risk factors associated with cardiovascular diseases among adults in Taif city, Kingdom of Saudi Arabia. More so, the cross-sectional study identifies the overall point prevalence of the significant lifestyle-related CVD factors, including smoking, physical activity, and dietary risks among the adults in Taif city, as well as the demographic and lifestyle correlates to use in formulating future population-specific health promotional campaigns and educational crusades.

## Materials and methods

This study adopted a cross-sectional survey design to determine the level of lifestyle-related risk factors for cardiovascular diseases among adult patients in Taif city, Saudi Arabia. The cross-sectional design is suitable for identifying the prevalence and its related factors at a particular period in the target population. It is crucial to determine the present cardiovascular risk factors existing in the intended population group. A multistage stratified sampling technique has been used to ensure that the sample is representative of the target population. In the first stage of sampling, the Private Healthcare Systems (PHCs) in Taif city were divided into two main categories according to their location (either inside or outside Taif city). There are 19 centers inside Taif and 97 centers outside Taif. In the second stage, four PHCs were selected randomly from each selected category, four centers inside Taif city and four outside Taif, and the sample size was proportionally allocated between them according to the served population in each center. In the third stage, the predetermined PHC sample selected was randomly selected using a systematic random sampling technique. The final sample size comprised 300 adult participants, representing the population served by these PHCs. Moreover, the inclusion criteria are adult patients aged 18 years and above who regularly attended the selected PHCs in Taif city and are willing to participate. However, individuals under 18 years of age, those who have been diagnosed with cardiovascular disease, or those unwilling to provide informed consent were excluded from the study. The key variables of the study are as follows: disease history, which refers to any illness prior to the onset of cardiovascular diseases (CVDs); smoking history, categorized into smokers and nonsmokers; body mass index (BMI), classified as underweight, normal, overweight, or obese; physical activity, determined by whether individuals engage in regular exercise; dietary habits, assessed based on high consumption of fatty foods; job, categorized as one or more occupations; and job stress, based on whether the job is considered stressful.

The data were gathered by a survey questionnaire adopted from Jafary et al. and Ahmed et al. [11,12]. The questionnaire was administered to participants at the PHC centers during scheduled visits, ensuring data collection occurred in a clinical setting. The information sought by this questionnaire includes demography, smoking history, exercise, diet, and understanding of risk factors of cardiovascular disease. Among the quantitative methods used to describe the study population, descriptive statistics were completed, to sum up the measures of risk factors and demographics. All statistical analyses were done using statistical software, SPSS version 20 (Armonk, NY: IBM Corp.). In addition, it must be mentioned that all the findings have been collected and analyzed in compliance with the principles of ethical practice. Participants' consent has been sought for the project, and all their information remains confidential. The participants were free to withdraw from the study at any given time.

## Results

The demographic profile of the sample in Table 1 shows a nearly equal distribution of gender, with 51.0% male and 49.0% female participants. The age groups are fairly balanced, with 26.0% under 30 years, 27.0% between 30 and 40 years, and 20.0% between 40 and 50 years, while 27.0% are above 50 years. Marital status is also evenly split between married (51.0%) and single/other (49.0%). Regarding religion, 70.7% of participants are Muslim, while 14.7% identify as Christian or other religions. The majority (59.0%) reside in urban areas, while 41.0% from rural regions. In terms of education, the largest group completed higher school education (31.0%), followed by those with secondary (26.0%) and university education (25.0%). In terms of occupation, 38.0% are semiskilled, while skilled and unskilled workers make up 29.0% and 33.0%, respectively. The largest income group earns between 2001 and 3000 riyals (39.3%), with the next largest group earning more than 3000 riyals (37.3%). Regarding lifestyle factors, 42.0% report work-home stress, while 55.0% have experienced depression in the past 12 months. The majority of participants (49.3%) are classified as obese based on their BMI, and a large percentage (72.3%) are smokers. A smaller portion (34.3%) consumes alcohol, and 78.7% do not regularly consume fried foods. Notably, 62.7% hold more than one job, and 63.0% report that their jobs require sleep deprivation. A high proportion (80.7%) engage in regular exercise and 56.0% report job-related stress. Lastly, 53.0% feel consistently anxious or nervous, and 30.0% report drug or substance use.

**Table 1:** Summary of demographic indicators.

Variables	Category	Count (n)	Percentage (%)
Gender	Male	153	51.0
	Female	147	49.0
Age group	Below 30 years	78	26.0
	30-40 years	81	27.0
	40-50 years	60	20.0
	Above 50 years	81	27.0
Marital status	Married	153	51.0
	Single/other	147	49.0
Residence	Urban	177	59.0
	Rural	123	41.0
Education	Secondary education	78	26.0
	Higher school education	93	31.0
	Technical education	54	18.0
	University education	75	25.0
Occupation	Semiskilled	114	38.0
	Skilled	87	29.0
	Unskilled	99	33.0
Income group	Less than 1000 riyals	29	9.7
	1001-2000 riyals	41	13.7
	2001-3000 riyals	118	39.3
	More than 3000 riyals	112	37.3
Work-home stress	Yes	126	42.0
	No	174	58.0
Depressed in the past 12 months	Yes	165	55.0
	No	135	45.0
Illness before disease	None	102	34.0
	Minor illness	93	31.0
	Major illness	105	35.0
BMI	Underweight	53	17.7
	Normal weight	50	16.7
	Overweight	49	16.3
	Obese	148	49.3
Smokes	Yes	217	72.3
	No	83	27.7
Consumes fried foods	No	236	78.7
	Yes	64	21.3
Employment (more than 1)	Yes	188	62.7
	No	112	37.3
Job requires lack of sleep	Yes	111	37.0
	No	189	63.0
Regular exercise	No	58	19.3
	Yes	242	80.7
Job stress	Yes	168	56.0
	No	132	44.0
Always anxious/nervous	Yes	159	53.0
	No	141	47.0
Drug/substance use	No	270	70.0
	Yes	30	30.0

Respondents were asked to rate various aspects of their cardiovascular health and related behaviors, as shown in Table 2. When asked to rate their overall cardiovascular health, the average score was 2.36 out of 5, with 32.0% rating their health at 2 and 30.0% at 3, indicating a generally moderate perception of cardiovascular health. A similar pattern is observed when participants were asked how often they experience symptoms like chest pain or shortness of breath during physical activity, with an average score of 2.27. Most responses clustered around 1, 2, and 3 (88, 86, and 93 participants, respectively), showing frequent experiences of mild symptoms. When rating their ability to perform daily physical activities without discomfort, the average score was 2.25, with the highest proportion (32.7%) rating themselves at 1, indicating limitations in physical activity for many participants. In terms of monitoring cardiovascular health, the average score was 2.37, with responses spread across 1 and 2 and reflecting a moderate tendency to check health markers like blood pressure or cholesterol. Satisfaction with cardiovascular health management had a similar mean of 2.37, with 27.0% of participants giving a rating of 1, reflecting a generally low level of satisfaction with their health management strategies. These responses indicate that while participants perceive moderate cardiovascular health, many experience symptoms and limitations in daily activities, and there is room for improvement in health management practices.

**Table 2:** Cardiovascular diseases among adults.

Questions	Rating	Frequency (count)	Percentage	Mean	Standard deviation
How would you rate your overall cardiovascular health?	1	74	24.7%	2.36	1.08
	2	96	32.0%		
	3	90	30.0%		
	4	27	9.0%		
	5	13	4.3%		
How often do you experience symptoms such as chest pain, shortness of breath, or fatigue during physical activity?	1	88	29.3%	2.27	1.06
	2	86	28.7%		
	3	93	31.0%		
	4	24	8.0%		
	5	9	3.0%		
How would you rate your ability to perform daily physical activities without experiencing cardiovascular-related discomfort?	1	98	32.7%	2.25	1.10
	2	76	25.3%		
	3	89	29.7%		
	4	28	9.3%		
	5	9	3.0%		
How frequently do you check or monitor your cardiovascular health (e.g., blood pressure, cholesterol levels)?	1	77	25.7%	2.37	1.09
	2	87	29.0%		
	3	95	31.7%		
	4	29	9.7%		
	5	12	4.0%		
How satisfied are you with your current cardiovascular health management (e.g., diet, exercise, medication)?	1	81	27.0%	2.37	1.13
	2	86	28.7%		
	3	88	29.3%		
	4	30	10.0%		
	5	15	5.0%		

Table 3 demonstrates that the CVD score shows a mean of 0, with a standard deviation of 1, indicating the data have been standardized. Work-home stress has a mean score of 1.58, suggesting a moderate level of stress experienced by participants. Other significant findings include depression in the past 12 months, with a mean of 1.45, indicating that slightly less than half of the participants experienced depression. BMI shows a mean of 2.97, reflecting a higher prevalence of overweight and obesity in the sample. High rates of smoking (mean 1.28) and drinking alcohol (mean 1.34) are also notable, as are lower levels of regular exercise (mean 1.81) and job-related stress (mean 1.44).

**Table 3:** Descriptive statistics (n=300). CVD: cardiovascular disease

Variables	Mean	Standard deviation
CVD score	0.0	1.0
Work home stress frequency	1.58	0.494
Depressed in past 12 months	1.45	0.498
Illness before disease	2.01	0.832
BMI	2.97	1.171
Smokes	1.28	0.448
Drinks alcohol	1.34	0.476
Consumes fatty foods	1.21	0.410
Holds more than one job	1.37	0.484
Job requires lack of sleep	1.63	0.484
Regular exercise	1.81	0.396
Job stress	1.44	0.497
Always anxious/nervous	1.47	0.500
Drug substance use	1.50	0.501

The multivariate model summary in Table 4 explains that the model explains 19.6% of the variance in the CVD score (R square=0.196). The adjusted R square value of 0.159 suggests a slight decrease in explanatory power after accounting for the number of predictors. The model’s overall significance (p=0.0) indicates that the predictors, when taken together, significantly impact CVD scores.

**Table 4:** Model summary of multivariate analysis. ^a^Dependent variable is the CVD score. Model 1: the first model tested; R: multiple correlation coefficient; CVD: cardiovascular disease; df: degree of freedom; F: F-statistic used to assess overall model significance

Model		R	R square	Adjusted R square	Standard error of the estimate	Change statistics					Durbin-Watson test
						R square change	F change	df1	df2	Significant F change^a^	
1	Regression	0.442	0.196	0.159	0.91708796	0.196	5.347	13	286	0.0	1.791

The ANOVA in Table 5 supports the significance of the model, with an F-value of 5.347 and a p-value of 0.0, confirming that the regression model is a good fit for predicting CVD scores. The sum of squares for the regression model (58.46) compared to the residual (240.54) shows that the model captures a meaningful portion of the variation in CVD outcomes.

**Table 5:** ANOVA estimates of multivariate analysis. ^a^Dependent variable is the CVD score. Model 1: the first model tested; R: multiple correlation coefficient; CVD: cardiovascular disease; df: degree of freedom; F: F-statistic used to assess overall model significance

Model		Sum of squares	df	Mean square	F	Significance
1	Regression	58.46	13	4.497	5.347	0.0^a^
	Residual	240.54	286	0.841		
Total		299	299	-		

Table 6 depicts the coefficient estimates of multivariate analysis that affirms that several predictors significantly contribute to the CVD score. BMI has a negative coefficient (-0.116), indicating that higher BMI is associated with worse cardiovascular health (p=0.017). Consuming fatty foods (B=0.271, p=0.047), holding more than one job (B=0.320, p=0.008), and job stress (B=0.212, p=0.064) are positively associated with CVD risk. In contrast, lack of sleep due to work (B = -0.325, p = 0.006) and regular exercise (B = -0.575, p = 0.000) are negatively associated with the CVD score, indicating protective effects. Other variables, like smoking, alcohol consumption, and anxiety, did not show statistically significant associations.

**Table 6:** Coefficient estimates of multivariate analysis. B: coefficient; t: t-statistic; CVD: cardiovascular disease

Variables	Unstandardized coefficients		Standardized coefficients	t	Significance	95.0% Confidence interval for B	
	B	Standard error	Beta			Lower bound	Upper bound
Constant	1.130	0.590		1.916	0.056	-0.031	2.291
Work home stress frequency	-0.036	0.112	-0.018	-0.324	0.746	-0.256	0.183
Depressed in past 12 months	-0.027	0.109	-0.013	-0.244	0.807	-0.242	0.189
Illness before disease	-0.043	0.067	-0.036	-0.651	0.516	-0.175	0.088
BMI	-0.116	0.048	-0.136	-2.405	0.017	-0.211	-0.021
Smokes	-0.082	0.134	-0.037	-0.612	0.541	-0.345	0.181
Drinks alcohol	0.202	0.126	0.096	1.602	0.110	-0.046	0.451
Consumes fatty foods	0.271	0.136	0.111	1.996	0.047	0.004	0.538
Holds more than one job	0.320	0.119	0.155	2.688	0.008	0.086	0.555
Job requires lack of sleep	-0.325	0.116	-0.157	-2.790	0.006	-0.554	-0.096
Regular exercise	-0.575	0.142	-0.227	-4.054	0.000	-0.854	-0.296
Job stress	0.212	0.114	0.106	1.862	0.064	-0.012	0.437
Always anxious nervous	-0.077	0.109	-0.038	-0.699	0.485	-0.292	0.139
Drug substance use	-0.109	0.110	-0.054	-0.990	0.323	-0.325	0.107

Table 7 presents the relationship between various demographic factors and the prevalence of cardiovascular disease (CVD) among adults. Gender does not significantly affect CVD, as indicated by a chi-square value of 0.18 and a p-value of 0.675. Similarly, marital status, religion, residence, education, occupation, and income group also show no significant association with CVD risk, with p-values above 0.05. However, the age group shows a statistically significant association with CVD (chi-square=23.18, p=0.000037). Notably, adults over 50 years of age have a higher prevalence of low CVD, while younger individuals (below 30 years) have a higher prevalence of high CVD. This indicates that age is a key factor influencing cardiovascular health in this population. Other variables do not demonstrate a statistically significant relationship with CVD risk.

**Table 7:** Factors related to cardiovascular disease among adults (n=300). CVD: cardiovascular disease

Variable	Category	Low CVD (n)	High CVD (n)	Chi-square	p-Value
Gender	Male	89	64	0.18	0.675
	Female	81	66		
Age group	Below 30 years	35	43	23.18	0.000037
	30-40 years	41	40		
	40-50 years	30	30		
	Above 50 years	64	17		
Marital status	Married	83	70	0.56	0.456
	Single/other	87	60		
Religion	Islam	125	87	1.60	0.450
	Christianity	22	22		
	Other	23	21		
Residence	Urban	101	76	0.002	0.962
	Rural	69	54		
Education	Secondary education	44	34	0.32	0.956
	Higher school education	53	40		
	Collegiate education	29	25		
	University education	44	31		
Occupation	Semiskilled	69	45	1.12	0.571
	Skilled	47	40		
	Unskilled	54	45		
Income group	Less than 1000 Riyals	16	13	0.08	0.994
	1001-2000 Riyals	23	18		
	2001-3000 Riyals	68	50		
	More than 3000 Riyals	63	49		

## Discussion

The adoption of the Western diet is a consequence of the various economic transformations that the young population of Saudi Arabia is experiencing. This has therefore contributed to the increase in the prevalence of CVD leading to a huge burden on the health systems. However, there is a lack of basic knowledge regarding the risk indicators of CAD. This means that as awareness is raised in the public domain, it also increases awareness and practices that are likely to help prevent CVD. It has also been found that there is an important relationship between health promotion behaviors and the knowledge of heart disease. In the same context, Al-Hazzaa has confirmed that more than half of the Saudi population engages in insufficient physical activity [13]. The observed low level of physical activity in Taif city may be associated with sedentary lifestyle changes brought about by urbanization, the rise of technology-focused activities, and cultural beliefs that restrict women from participating in exercise regimes outside the home. This is a rather worrisome sign since physical inactivity is known to be one of the determinants of CVDs and can lead to obesity, high blood pressure, and pre-diabetes. Besides, poor diet and lack of exercise are the main causes of hospitalization related to heart diseases, cancer, and diabetes, occupying three-quarters of all cases. Saudis are said to be physically inactive, and the fast-food diet includes high fats, saturated fats, sodium, carbohydrates, and low fiber [10]. Thus, it can be stated that the participants had a highly lipophilic diet containing a high amount of saturated fats, sugars, and salt, while their consumption of fruits and vegetables was low. These findings support literature reviews that pointed to the nutritional deterioration in Saudi Arabia from a traditional diet to a more Western diet [14]. The findings on Taif city reveal that adults have a high proportion of poor dietary habits, which is shown to be a modifiable risk factor for the development of CVDs. Adequate nutrient intake and the avoidance of excessive sodium and calories through the consumption of a higher intake of fruits, vegetables, and whole grains can help in the prevention of CVD.

As per one of the previous studies done on the consumption of fast foods and obesity, KSA has established a 5% CAD prevalence, which is halfway between other countries. Several classical CAD risk factors in the Saudi population include age ≥60 years, male gender, overweight, hypertension, smoking, diabetes mellitus, hypertriglyceridemia, and hypercholesterolemia [14]. Also, it was observed that the study participants had a high prevalence of obesity, a factor well known to increase CVD risks. Overweight and central obesity are known independent risk factors for CVD, as both conditions are associated with dyslipidemia, hypertension, and type 2 diabetes mellitus. These findings are also in line with the national data, which show that the levels of obesity in Saudi Arabia are alarming, especially among women. In the current study, physical activity was found to be relatively rare since only 48% of the participants were found to undertake any form of exercise. Therefore, more public health interventions for weight loss and healthy diet and physical activity are required to improve this situation. It has also been observed a rise in obesity, high cholesterol, and metabolic disorders among teenagers and young adults of the society over the last few decades.

From the study, it was also evident that Antigua and Barbuda had a high smoking rate, especially among men. Tobacco use, especially smoking, is known to be an independent risk factor for CVDs due to its effects on atherosclerosis, thrombosis, and endothelial dysfunction [15]. The current proportions of smoking observed among the participants of Taif city can be compared to the latest Saudi Arabian statistics, according to which smoking is still an important issue affecting the general and individual population’s health in the country [15]. Therefore, more effective strategies need to be designed, and efforts have to be directed at young people and culturally appropriate messages.

Another common risk factor, as noted in this study, was hypertension, most of which was accompanied by obesity and/or diabetes. From the high prevalence rate of hypertension in Taif city, this study falls in line with the increased prevalence of hypertension in Saudi Arabia, recognized in the recent past as a source of morbidity and mortality. The presence of hypertension with other modifiable CVD risk factors like obesity and lack of exercise increases the need for multiple target interventions.

In light of the similar comprehensive investigations conducted in other areas of Saudi Arabia, the results obtained in Taif city are in line with the Saudi Arabia trend. For instance, a study conducted among adults in Riyadh to determine the prevalence of obesity, smoking, and hypertension showed similar results [16]. However, in Taif, the dietary patterns and inactivity levels were slightly poorer which may be attributed to differences in the degree of urbanization and cultural practices. This comparison proves that for the successful intervention of the risk factors of CVD, there is a need to consider the cultural perspective of the different regions in the formulation of public health strategies.

Most of the authors found that myocardial infarction is more common in males than in females [17]. These sex differences might be associated with the differences in the distribution of fat in the body, as pointed out by Campbell [18]. Unfortunately, the mortality rate from heart disease in women increases after menopause, but it is still lower than the mortality rate in men. Stating the fact, it is important to note that age is one of the key indicators of risk of myocardial infarction (MI). In the present study, most of the participants were elderly (over 50 years old), and they highlighted that as the participants age, the incidence of coronary heart disorders rises [17]. According to the American Heart Association (AHA) statistics (2006), 83% of people who die from coronary heart disease are 65 years or older. [12]. In the study conducted, the researchers were able to establish that hypertension and diabetes mellitus are some of the leading modifiable risk factors of MI. This result is concorded with findings obtained in the majority of previous research studies, including those of Malinauskiene and Kondo et al. [19,20]. Patients with hypertension are susceptible to coronary artery disease because hypertension applies extra pressure on the walls of arteries. As time goes on, such pressure will cause harm to the arteries. These injured arteries are also prone to develop some degenerative changes that lead to the deposition of fatty materials that harden the arterial walls. Diabetes mellitus greatly enhances the occurrence of incidence and mortality to CVD attack and recurrence of CVDs in middle-aged persons. This was consistent with many other previous studies, where it was found that 71.8% of the participants in the present study were smokers. Tachycardia, which is a result of cigarette smoking, puts more work on the heart, thus reducing its efficiency [21]. Moreover, smoke contains nicotine, which is toxic to the human body as it triggers the release of adrenaline, causing increased heart rate and blood pressure, hence increasing the workload on the heart and, thus, the rate of coronary heart diseases.

As found in the present study, fried food was perceived as an important risk factor for developing myocardial infarction [22,23]. Some previous research studies explain that the frying process alters the quality of food and might raise the amount of fat and trans fatty acids in food which would be why fried food puts people at high risk of myocardial infarction. Most of the participants (89.7%) in this study had a sedentary lifestyle. According to Bonde et al., repetitive and heavy lifting or carrying at work and strenuous physical workload were associated with acute CVDs [24]. And they discovered a lower risk of CVD for the active subjects as compared to the passive subjects. Given the fact that a third of the population in Taif city is at risk of CVDs due to the increased rates of lifestyle-related risks, there is a need for an intervention. These should comprise programs addressing physical activity, dietary habits, smoking iniquities, and health check-ups within the community. Furthermore, policymakers need to think about the cultural and socio-economic factors that may determine health behavior in this part of the world. The burden of CVDs is increasing, and therefore, it requires a complex intervention involving key stakeholders, such as clinicians, the chiefs, and community health workers [24-26].

Ruan et al. and Ogunmola et al. point out socio-economic and healthcare inequalities for cardiovascular diseases in low- and middle-income countries (LMICs) since a majority of such patients have poor access to preventive and curative care [27,28]. On the other hand, Yang et al. and Hamer et al. focus on more urbanized and advanced locations, exploring factors linked to lifestyles, such as malnutrition, and lack of exercise, and their combination with other health crises, including coronavirus disease 2019 [29,30]. The end result points to a clear pattern in which the concentration of at least one of many risk factors requires identified strategies that consider socio-ecological determinants to address the problem.

## Conclusions

This cross-sectional study on the demographic and socio-clinical characteristics of adults in Taif city, Saudi Arabia, about lifestyle-related risk factors of CVDs, yields several significant findings. First, demographical assessment proved that the sex, age, and educational attainment ratios of participants provided a diverse sample of people. The findings suggest high levels of CVD risk factors, which include smoking, hypertension, diabetes, poor diet, lack of physical activity, and stress among the adults in Taif city. For instance, over 50% of the patients reported having work or home stress, many engaged in smoking, and had other problems, including high blood pressure, diabetes, or high lipid diets. Promoting the awareness of healthy lifestyles, stress-free lifestyles, cessation of smoking, and regular practice are some of the most useful tools in preventing CVDs in this group of people. Further studies should embrace studies with greater analytic design to establish the necessary sequential relationships and efficiency of curative measures in lowering the CVD threat among the identified population.
